# Dissection of the anti-*Candida albicans* mannan immune response using synthetic oligomannosides reveals unique properties of β-1,2 mannotriose protective epitopes

**DOI:** 10.1038/s41598-021-90402-4

**Published:** 2021-05-24

**Authors:** Boualem Sendid, Karine Lecointe, Mayeul Collot, Pierre-Marie Danzé, Sébastien Damiens, Anne-Sophie Drucbert, Chantal Fradin, Jean-Pierre Vilcot, Frédéric Grenouillet, Faustine Dubar, Jérôme de Ruyck, Samir Jawhara, Jean-Maurice Mallet, Daniel Poulain

**Affiliations:** 1 INSERM U1285, Univ. Lille, CHU de Lille, UMR CNRS 8576 - UGSF - Unité de Glycobiologie Structurale et Fonctionnelle, 59000 Lille, France; 2 École Normale Supérieure, Département de Chimie, Laboratoire des Biomolécules, UMR CNRS 7203, 24 rue Lhomond, 75005 Paris, France; 3 Institut de Biochimie, CHU de Lille, 59037 Lille Cedex, France; 4 Univ. Lille, INSERM, CHU Lille, Pasteur Institute of Lille, U1167 - RID-AGE, 59000 Lille, France; 5 Institut D’Électronique de Microélectronique et de Nanotechnologie (IEMN), UMR 8520, Université de Lille, Villeneuve d’Ascq, France; 6 Department of Parasitology-Mycology, University Hospital, Besançon, France

**Keywords:** Biochemistry, Chemical biology, Immunology, Microbiology

## Abstract

*Candida albicans* mannan consists of a large repertoire of oligomannosides with different types of mannose linkages and chain lengths, which act as individual epitopes with more or less overlapping antibody specificities. Although anti-*C. albicans* mannan antibody levels are monitored for diagnostic purposes nothing is known about the qualitative distribution of these antibodies in terms of epitope specificity. We addressed this question using a bank of previously synthesized biotin sulfone tagged oligomannosides (BSTOs) of α and β anomery complemented with a synthetic β-mannotriose described as a protective epitope. The reactivity of these BSTOs was analyzed with IgM isotype monoclonal antibodies (MAbs) of known specificity, polyclonal sera from patients colonized or infected with *C. albicans*, and mannose binding lectin (MBL). Surface plasmon resonance (SPR) and multiple analyte profiling (MAP) were used. Both methods confirmed the usual reactivity of MAbs against either α or β linkages, excepted for MAb B6.1 (protective epitope) reacting with β-Man whereas the corresponding BSTO reacted with anti-α-Man. These results were confirmed in western blots with native *C. albicans* antigens. Using patients’ sera in MAP, a significant correlation was observed between the detection of anti-mannan antibodies recognizing β- and α-Man epitopes and detection of antibodies against β-linked mannotriose suggesting that this epitope also reacts with human polyclonal antibodies of both specificities. By contrast, the reactivity of human sera with other α- and β-linked BSTOs clearly differed according to their colonized or infected status. In these cases, the establishment of an α/β ratio was extremely discriminant. Finally SPR with MBL, an important lectin of innate immunity to *C. albicans*, classically known to interact with α-mannose, also interacted in an unexpected way with the protective epitope. These cumulative data suggest that structure/activity investigations of the finely tuned *C. albicans* anti-mannose immune response are worthwhile to increase our basic knowledge and for translation in medicine.

## Introduction

Many studies have demonstrated that molecules essential for normal cell physiology are usually glycosylated and variations in their glycosylation patterns often induce changes in their function. These changes have been reported in both prokaryotic and eukaryotic organisms^1^. In fungi, oligosaccharides are important molecules playing a crucial role in fungal cell–cell communication and host–pathogen interactions involving innate immunity receptors. Conjugated to proteins or lipids, oligosaccharides elicit strong antibody responses as a result of fungal tissue invasion. From a more general point of view, as they have a high level of specificity, anti-oligosaccharide antibodies are widely used as a tool for blood grouping, control of vaccination status, or serotyping of viral and bacterial agents^2, 3^.

The *C. albicans* cell wall is a multilayered structure composed mainly of sugars (> 80%) and to a lesser extent proteins and lipids. The surface is covered by phosphopeptidomannan (commonly named mannan), a high molecular weight matricial component, expressing a large repertoire of α- and β-oligomannosides. PPM is interwoven with numerous mannoproteins (MPs) emerging at the cell surface and covalently bound to the inner layer composed of glucans. These mannoproteins are distributed in GPI anchor or PIR proteins depending on their type of linkage to glucans. Of note, these MPs display the same oligomannose repertoire as PPM^4^. The cell wall surface is also covered by a glycolipid with β-mannose epitopes known as phospholipomannan (PLM). The intermediate layer of the cell wall is composed of a polysaccharide polymer backbone consisting of β-glucans (both β-1,3, and β-1,6 D-glucopyrannosyls) while a chitin matrix participates in the hydrophobic layer of the cell membrane^5^. From an immunological point of view, mannans and β-glucans are differently recognized by host pattern recognition receptors (PRRs). β-1,3-glucans are recognized by dectin-1, α-mannan is recognized by the mannose receptor, dectin-2, DC-SIGN, mannose-binding lectin (MBL), and langerin, whereas β-mannans are specifically recognized by galectin-3^6^.

Anti-glycan/oligosaccharide antibodies are also used as biomarkers for the diagnosis of human invasive^7^ or allergic^8^ fungal diseases. Anti-oligosaccharide antibody specificity is conferred by the nature of the constitutive sugar sequences, the type of linkage and the length of the oligosaccharide chain^9^. For immunoassays, these carbohydrate antigens need to be immobilized on the surface of microtiter plates or magnetic microspheres, which represents a challenging task. This pitfall has been overcome by using synthetic oligosaccharides, which can be prepared with a versatile anchor group. Biotin is the most widely used of these as it selectively binds to a large number of surfaces. Such tagged oligosaccharides are strongly recognized by avidin and are successfully used in immunoassays^10^.

For experimental purposes, surface plasmon resonance (SPR) has been used to decipher the interactions between oligosaccharides and proteins or peptides. Such investigations were performed to characterize lectin-microbial interactions. MBL is a multimer of polypeptide chains 32 kDa in size. Three polypeptide chains make up a triple helix with a collagenous region^11^, which is the basic circulating subunit of MBL. In serum, MBL is composed of oligomers of subunits from dimers to hexamers, which are effector forms of the lectins for glycan or pathogen interactions^12, 13^. MBL interacts with terminal-D-mannose residues, L-fucose, and GlcNAc^14, 15^ but also with bacteria, virus, molds, yeasts, and parasites. It is known that MBL can recognize *C. albicans*^16^ but the minimal epitope necessary for this recognition is unknown.

The main objectives of the present study were to use synthetic oligosaccharides to carry out epitope mapping of the main monoclonal antibodies (MAbs) directed against the cell wall of *C. albicans*, to identify the oligomannoside epitopes recognized by MBL through SPR, and to dissect the anti-mannan antibody response in the serum of patients with invasive candidiasis using multi-analyte profiling (MAP) technology^10^. To reach these objectives a bank of previously synthesized biotin sulfone tagged oligomannosides (BSTOs) of α and β anomery^10^ was complemented with the synthetic β-mannotriose described as a protective epitope. This revealed unexpected results regarding previous paradigms of *C. albicans* mannose epitope recognition.

## Results

### MAP analysis of BTSO reactivity with anti-carbohydrate MAbs

**Figure 1:**
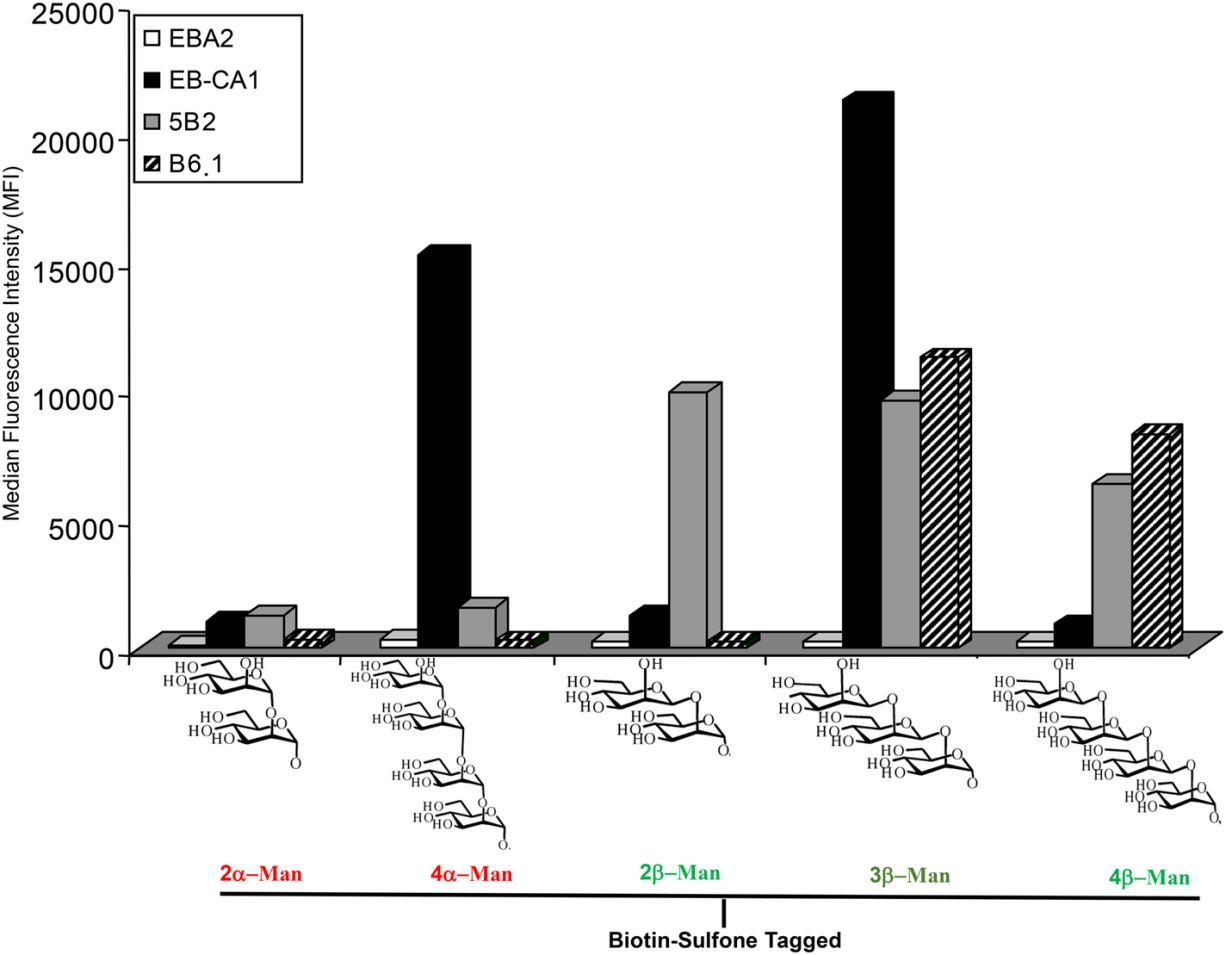
Multi-analyte profiling (MAP) analysis of biotin sulfone tagged synthetic oligomannoside (BTSO) reactivity with anti-carbohydrate monoclonal antibodies (MAbs). Results are shown as the mean fluorescence intensity obtained with MAbs (EB-CA1: black bars; 5B2: grey bars; and B6.1: hatched bars), or EB-A2 (white bars) for each microbead set coated with different BSTOs including α-1,2-mannobioside (2α-Man), α-1,2-mannotetraose (4α-Man), β-1,2-mannobioside (2β-Man), β-1,2-mannotriose (3β-Man), and β-1,2-mannotetraose (4β-Man).

Five BSTO epitopes were individually coupled to different batches of microsphere beads and tested with the panel of anti-*C. albicans* MAbs. As shown in Fig. 1, the anti-galactofuran MAb (EB-A2) did not generate any signal with any of the mannose residues presented as BSTOs. As expected, MAb 5B2 bound selectively to members of the β-mannose family (β-Mans) with reactivity starting from mannobiose. MAb B6.1 reacted strongly with its specific β-mannotriose epitope and to a lesser extent with neighboring 3β-Man. MAb EB-CA1, described as reacting with 5α-Man, bound to 4α-Man, which was the closest epitope in our series of BSTOs, but did not react with 2β-Man or 4β-Man. Unexpectedly, MAb EB-CA1 displayed high reactivity with 3β-Man providing the first exception, so far, to the α/β Man dichotomy of anti-*C. albicans* antibody reactivity.

### SPR analysis of BSTO reactivity with anti-carbohydrate MAbs

**Figure 2:**
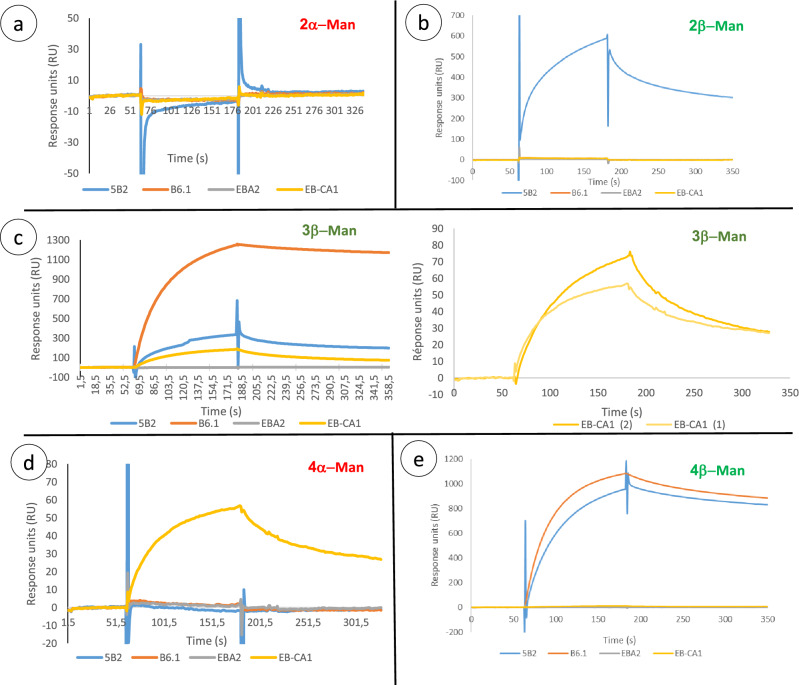
Surface plasmon resonance analysis of biotin sulfone tagged synthetic oligomannoside (BSTO) reactivity with anti-carbohydrate monoclonal antibodies (MAbs). MAbs B6.1 (orange curve), 5B2 (blue curve), EB-CA1 (yellow curve), and control (EB-A2, grey curve) were successively tested with 2α-Man (**a**), 2β-Man (**b**), 3β-Man (**c**), 4α-Man (**d**), and 4β-Man (**e**).

The interactions between ligand BSTOs and MAbs are shown in Fig. 2. Figure 2a shows an example of the absence of interaction concerning 2α-Man where changes to baseline are only due to variations in the injected buffer and the signal is stable. The functionality of the 2α-Man sensor was confirmed by reactivity with ConA (250 nM) (data not shown). Variations of about 5 RU for MAb 5B2 during the injection step are negligible and modifications of sensorgram profiles can only be attributed to variations in buffer composition. A comparison of relative affinity was performed at 250 nM for each antibody, and in the presence of binding two steps can be observed. During the injection step, the association can be seen as an exponential phase. Typical results of the interaction between BSTOs and antibodies are shown in Fig. 2b–e.

MAb EB-CA1 weakly interacted with 4α-Man (~ 60 RU) and presented a typical profile of a sensorgram with association and dissociation curves (beginning after 60 s and 180 s, respectively). Surprisingly, EB-CA1 showed specific interaction with 3β-Man (Fig. 2c) and the amplitude of the signal was similar to that obtained with 4α-Man (Fig. 2d). The unexpected reactivity of EB-CA1 with 3β-Man was confirmed by replications of injections (3c, right panel). EB-A2, B6.1, and 5B2 MAbs did not show any interactions with 4α-Man.

Interactions of antibodies with β-Mans were more informative. Only MAb 5B2 interacted with 2β-Man (~ 600 RU) and 4β-Man (~ 800 RU) (Fig. 2b, e). These results can also be observed for 3β-Man in a less important manner (~ 300 RU) (Fig. 2c left panel). There was no interaction with 2α-Man and 4α-Man (Fig. 2a, 3d). MAb B6.1 showed strong binding to 3β-Man and 4β-Man. The absence of response with EB-A2 injections used as a control on each sensorgram confirms the specificity of the binding with the other antibodies. Saturability of the interactions was not performed because of the IgM nature of the antibodies which have 10 binding sites per molecule.

**Figure 3:**
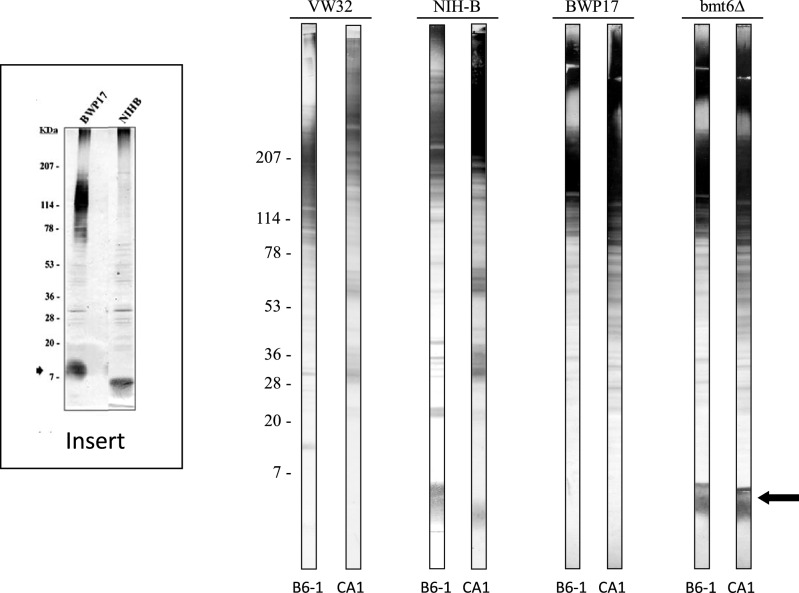
Western blots of whole cell extracts from *C. albicans* serotype A (VW32, BWP17 and *bmt6Δ*) and serotype B (NIH B-792) strains probed with MAbs B6.1 and EB-CA1. The insert shows a serotype A strain (BWP17) and serotype B strain (NIHB) probed with MAb 5B2 showing the shift in *r*MW previously characterized structurally, and resulting from shortening of β-Man chain to 3 mannose residues in serotype B strains^34^.

Data obtained by SPR confirmed those observed with MAP in terms of differential binding conditioned by anomery and oligomannoside chain length. The only discrepancy concerned the amplitude of signals between the two technologies. Overall, the MAP and SPR results confirmed the reactivity of MAb EB-CA1 with 3β-Man as the first real exception observed so far to the specificity of anti-mannoside antibodies conferred by their anomeric configuration. It is not the specificity of the “protective” antibody that is questionable (B6.1), but the ability of the “protective epitope” to react with both anti-β-mannoside antibodies and anti-α-mannoside antibodies. We then aimed to confirm these data using native antigens from *C. albicans* known to express the protective epitope.

### Western blot analysis of EB-CA1 and B6.1 MAb reactivities against mannoglycoconjugates from *C. albicans* serotype A and B strains

In order to confirm the reactivity of the 3β-Man "protective epitope" with an anti-α-Man antibody western blot experiments were performed involving an extensively characterized molecule, PLM^18^. PLM is a member of the manno-inositol phosphoceramide (MIPC) family with an apparent *r*MW of 14–18 kDa in western blots, and with a polysaccharide moiety composed of long linear chains of β-Man in *C. albicans* serotype A strains^19^ (Fig. 3 insert, lane BWP17). Later, it was discovered that this cell wall surface molecule differed in *C. albicans* serotype B strains^20^ with a *r*MW of approximately 7 kDa (Fig. 3 insert Lane NIH B). Structural studies established that this shift in rMW was related to a truncated glycan moiety that corresponded to a trimannoside. This truncation was reproduced by inactivation of β-mannosyl transferase 6 (*BMT6*), responsible for the addition of the third β-mannose in serotype A^21^. Natural and genetically constructed variants of PLM therefore seemed particularly well adapted to confirm the unexpected reactivity of the protective epitope with MAb EB-CA1 (Fig. 3). When these extracts were probed with B6.1 and EB-CA1 MAbs, reactivity was observed with numerous of bands of *r*MW > 40 kDa in accordance with previous data that showed that both epitopes are distributed over a large number of mannoproteins that display a polydisperse character in the gel as a function of the increased molecular weight and increased size of their reactive polysaccharide moiety. Western blots performed with control MAb EB-A2 did not show any reactivity. Among the *C. albicans* glycoconjugates, we focused our analysis on PLM (Fig. 3, arrow). PLM of *C. albicans* serotype A VW32 and BWP17 strains had no reactivity with MAb B6.1 in contrast to *C. albicans* serotype B NIH B-792 and *C. albicans* serotype A *bmt6Δ* strain where deletion of both alleles of *BMT6* resulted in a truncated PLM with an accumulation of 3β-Man. Interestingly, MAb EB-CA1, specific for 4α-Man, displayed the same reactivity as MAb B6.1 with 3β-Man. Additional information concerning the technical procedure of WB used for generating Fig. 3 is available in the supplementary information section with specific references.

### Analysis of human antibody response with anti-mannan ELISA test and MAP involving different BSTOs for their differential ability to discriminate between controls, colonized, and *Candida*-infected patients

Systemic *Candida* infection is generally associated with a sharp rise in anti-*C. albicans* mannan antibodies leading to titers rarely encountered in non-infected patients. In parallel, mannanemia, (i.e. the release of mannan into patients’ sera resulting from host tissue invasion by *Candida*) may also be detected. On this basis, it has been proposed to combine the detection of anti-*C. albicans* mannan antibodies and mannanemia as a diagnostic strategy to compensate for the poor sensitivity of blood cultures.

**Figure 4:**
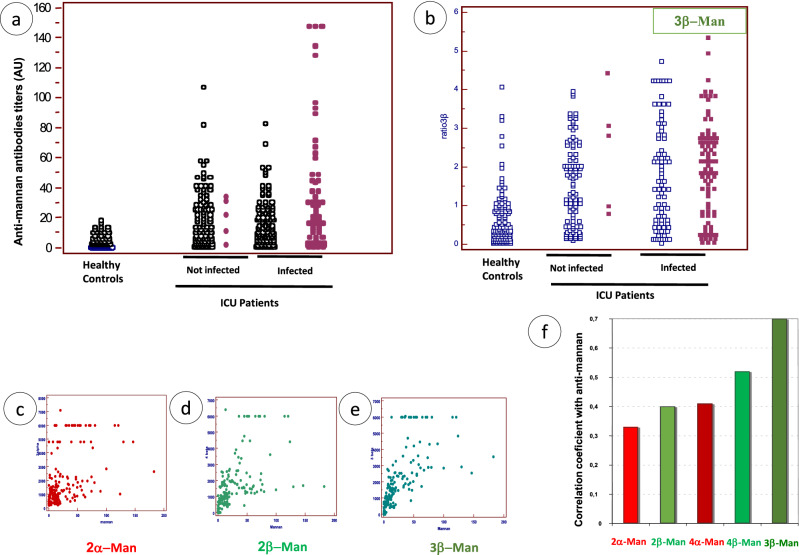
Reactivity of human sera against *C. albicans* mannan and BSTO. Reactivity against *C. albicans mannan* (**a**) as detected by ELISA (Platelia *Candida* Ab test), and against BST-3β-Man (**b**), as detected by multi-analyte profiling (MAP) analysis. Human sera consisted of sera from healthy controls (blood donors presenting no disease), and ICU patients either not-infected or infected with *C. albicans*. Anti-*C. albicans* mannan antibody response is shown for infected patients according to antigenemia status (negative: open symbols; positive: closed symbols) (**b**). The correlation between anti-mannan and 2α-Man, 2β-Man, 3β-Man antibodies is shown in (**c**, **d**, **e**) respectively. The values of Spearman rank correlation coefficients for the different BSTOs are given in f.

The results of the anti-*C. albicans* mannan antibody tests are plotted on Fig. 4a for each of the 181 sera taken from 30 infected intensive care unit (ICU) patients as well as the associated values for mannanemia. When considering the values for the same tests in non-infected ICU patients, a larger proportion than healthy controls also had high anti-mannan antibody titers in relation to the heavy colonization observed for most patients during hospitalization. A similar distribution was observed when considering the reactivity of human sera against 3β-Man (Fig. 4b).

Although different results could have been expected using 3β-Man as a single epitope, the results observed with infected patients' sera correlated with those obtained with mannan (Fig. 4e). When performing the same analysis with other BSTOs the results were heterogeneous by comparison to anti-mannan reactivity. Examples are given for the distribution of 2α-Man and 2β-Man in Fig. 4c, d, respectively, and the Spearman's rank correlation coefficients with mannan reactivity are shown as a function of the BSTO in Fig. 4f. Altogether these findings revealed the property of 3β-Man to mimic the mannan repertoire of β- and α-mannoside epitopes, and confirms its unique ability to bind both anti-β and anti-α mannose antibodies in human sera, as shown previously with MAbs in MAP and SPR.

**Figure 5:**
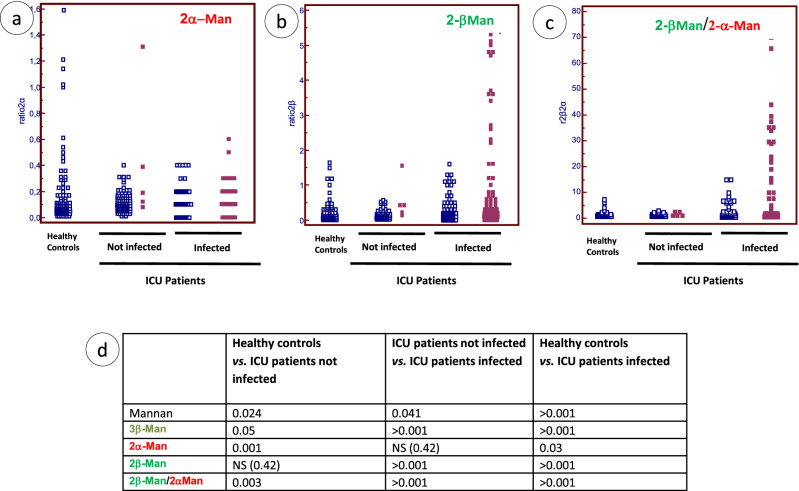
Reactivity of human sera against 2α-Man, 2-β-Man, and establishment of 2β-Man/2α-Man ratio for differentiation between uninfected and infected ICU patients. (**a**) 2α-Man, (**b**) 2β-Man and (**c**) 2βMan/2α-Man ratio (open squares: negative mannanemia; closed squares positive mannanemia). (**d**) Statistical analysis of the ability of different antigens to discriminate between study groups.

We then investigated whether other BSTOs would discriminate infected patients from other ICU patients (Fig. 5). Surprisingly, when using 2α-Man, reactivity decreased from controls to colonized and infected patients (Fig. 5a) (i.e. the converse of what would have been expected for identifying infected patients). By contrast, 2β-Man reactivity increased markedly in infected patients (Fig. 5b). Although these results were discriminatory per se we decided to determine the α/β reactivity ratio. As shown in Fig. 5c, more discrimination was observed between patients in relation to *C. albicans* saprophyte/pathogen transition.

The results of the statistical analysis of the ability of antigens to discriminate the different study groups are shown in Fig. 5d. The *p* values are reported for each comparison. 3β-Man and 2β-Man/2α-Man ratio provided the best discrimination whatever the categories compared. However major differences were observed depending on the groups under comparison with some antigens even giving non-significant results. This reveals the complex framework of *C. albicans* mannose repertoire recognition depending on the patient’s status.

### SPR analysis of BSTO-MBL interaction

**Figure 6:**
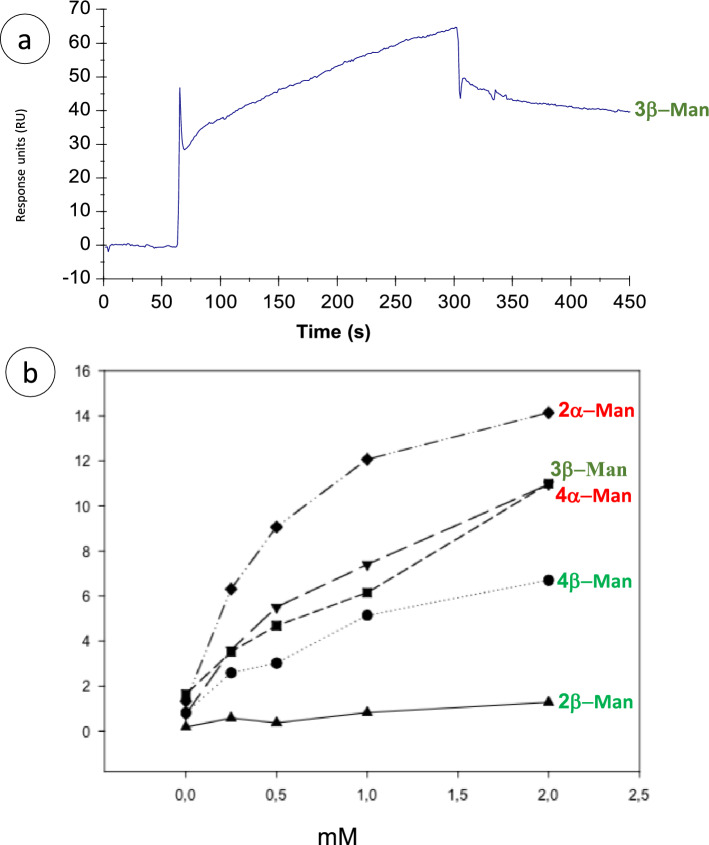
Affinity curves for recombinant mannose-binding lectin (rMBL) binding to oligomannosides. (**a**) Typical sensorgram of the interaction of MBL with 3β-Man (2 mM). (**b**) Normalized affinity curves of increasing mM of the different oligomannosides with MBL.

To further establish the interaction between oligomannosides and recombinant (r)MBL, SPR experiments were carried out using BIAcore 3000. An example of a typical sensorgram of the interaction is shown in Fig. 6a with 3β-Man. To characterize how the oligomannose anomery and degree of polymerization may influence binding to rMBL, the different oligomannosides were screened at 0, 0.25, 0.5, 1, and 2 mM (Fig. 6b). Although the interaction was characterized by low signals (< 100 RU), SPR sensograms clearly demonstrated differential interactions of BSTOs with rMBL.

As expected, for one MBL ligand already identified, specific binding occurred with 2α-Man. 4α-Man, which presents the same structure at its terminal non-reducing end, also displayed strong binding. Among the β-1,2 linked oligomannosides, 2β-Man displayed very low affinity, and although the 4β-Man signal was better, it was still weak and was not saturable even at high concentrations. Interestingly, the affinity curves for 3β-Man and 4α-Man were almost superimposable. These results were confirmed by regression curves displaying a correlation coefficient of > 0.9 and similar slopes (data not shown). Thus, these results obtained with a lectin of innate immunity sensing *C. albicans* PAMPs confirm the unique behavior of the 3β-Man "protective epitope" among the β-Man series observed with monoclonal and polyclonal antibodies.

## Discussion

The cell wall of *C. albicans* is a complex stratified and dynamic structure responsible for cell integrity and plays a pivotal role in host-yeast interactions. The cell wall is involved in adhesion, invasion, and is the target of innate and adaptive immune responses^6, 22^.

Among the cell wall polysaccharides, mannan is widely recognized to have an immunomodulatory effect^23–25^. Its structure has been well established by an impressive series of structural and serological studies from Suzuki's group^26, 27^. These authors established that *C. albicans* mannan is similar to the mannan of the closely related species *S. cerevisiae*, extensively studied as a model of glycosylation, with a comb-shaped structure composed of α-1,2, α-1,3, and α-1,6 mannose residues, but that *C. albicans*, *C. tropicalis* and *C. glabrata*, now reported as responsible for 90% of human *Candida* infections^28^, also have the ability to synthesize β1,2 mannose^4, 21, 29, 30^.

Simultaneously, at a time when yeast species identification was a lengthy procedure, Fukazawa's group^31^ developed an efficient *Candida* species identification kit (*Candida* Check; Iatron Laboratories, Tokyo, Japan). Cross-absorbed polyclonal rabbit antibodies were used for the identification of the main pathogenic *Candida* species by agglutination. The relevant antigen has been characterized as PPM and a series of seminal works about structure/activity relationships involving inhibition of agglutination together with nuclear magnetic resonance (NMR) and gas chromatography–mass spectrometry provided evidence that the antigenic determinants recognized by rabbit antisera are based on anomery of linkages (α-1,2, α-1,3, α-1,6, and β-1,2), and the degree of polymerization of mannose residues^32, 33^.

With the advent of hybridoma technology a relatively large number of anti-*C. albicans* MAbs have been produced which react against *C. albicans* mannan/mannoproteins^34–37^. Elucidation of their epitopes confirmed that they reacted with either anti-α-Man or anti-β-Man antibodies fitting the Iatron serological classification. Some of these MAbs were included in this study together with their BSTO epitopes. Due to interest in the protective effect of MAb B6.1 its epitope was specially constructed as a BSTO for this study.

In the present study, the specificity of these MAbs and polyclonal antibodies (human sera collected during *Candida* infection) was dissected using BSTOs in a qualitative and quantitative manner by SPR and MAP technology. We confirmed the specificity of 5B2 for 2β-Man^34^, B6.1 for 3β-Man^35^, and EB-CA1 for 4α-Man^38^. Unexpectedly, we observed that EB-CA1 also reacted with the 3β-Man epitope, which therefore represents the first exception to the dichotomous reactivity of mannan epitopes with either anti-α-Man or anti-β-Man antibodies established through a large number studies. This surprising observation, obtained by MAP and SPR, was confirmed by more conventional western blot analysis using *C. albicans* serotype B PLM and a serotype A deleted strain as both structurally defined antigens expressing the 3β-Man epitope. Studies by Cutler et al. emphasized the protective role of the humoral response directed against this epitope in both systemic and mucosal models of *Candida* infection^39^. A subsequent study showed that a monoclonal IgG3, called MAb C3.1, obtained from rabbits immunized with a liposome-mannan vaccine had the same specificity for β-mannotriose as MAb B6.1. Moreover, MAb C3.1 enhanced the resistance of mice to disseminated candidiasis^40^. Lipinski et al.^41^ developed a vaccine with 3β-Man and showed that it induced a strong secondary antibody response in rabbits. In addition, 3β-Man vaccination resulted in a reduction of the fungal burden in tissues of immunocompromised rabbits after challenge with live fungal cells. Another study combining NMR, chemical mapping, and computer simulation showed the importance of 3β-Man as the optimal oligosaccharide for the development of a vaccine against *C. albicans*^42^. In this study, importance was given to the anomery of linkage of the mannose at the reducing end, which is the case in our study for both the synthetic 3β-Man BSTO and the native 3β-Man-IPC from serotype B PLM. Despite experimental findings concerning the protection obtained with 3β-Man no translation was made in the analysis of the human response looking for protective antibodies targeting mannan epitopes. When BSTOs were used to dissect patient responses against individual epitopes in healthy individuals, colonized, or infected patients variable discrimination could be observed between these groups. For example, the 2α-Man response was higher in healthy controls than in patients. Conversely, 2β-Man antibodies were higher in infected patients, therefore representing a better diagnostic tool which was increased further by establishing the 2βMan/2α-Man ratio. These preliminary findings revealing the complex framework of *C. albicans* mannose repertoire recognition depending on patient status may have interesting clinical applications in terms of diagnosis and prediction of outcome. They certainly deserve further larger prospective studies combining MAP analysis and bioinformatic algorithms for the survey of hospitalized at-risk patients, particularly given that a recent study showed that patients infected with *Candida* strains displaying high levels of β1,2-mannosyl residues^43^ have a poor prognosis. With regard to 3β-Man, the significance of this response against a protective epitope cannot be evaluated in the absence of information on patient outcome. However, it had diagnostic value for discriminating colonized from infected patients. This property was shared with mannan as reflected by the Spearman correlation coefficient among the BSTOs. Thus, in coherence with results obtained with MAbs these findings revealed the property of 3β-Man to mimic the mannan repertoire of β- and α-mannoside epitopes.

MBL is a collectin with a molecular weight of 32 kDa, produced mainly by hepatocytes, that circulates in multimeric form with a predominance of the quaternary structure^44^. MBL forms a complex with three MBL-associated serine proteases^45^. MBL activates the lectin complement pathway after recognition of microorganisms through the carbohydrate-recognition domain. In vitro experiments using MBL purified from serum showed an interaction with the GlcNAc pentamer that activated the complement pathway. This interaction could be inhibited by mannose and N-acetylglucosamine. The carbohydrate-recognition domain of MBL senses polysaccharide patterns such as D-mannose, L-fucose, and GlcNAc on several clinically relevant pathogens including *C. albicans*. Lillegard et al.^16^ showed that depending on the growth conditions, *C. albicans* cells could fail to bind to MBL even though its cell wall contains mannose residues. They also reported variable patterns of MBL binding to yeast cells that are consistent with patterns of binding of anti-mannan MAbs to *C. albicans*. Epitopes recognized by some mannan specific MAbs are diffusely and continuously expressed on the surface of yeast cells (e.g. MAb EB-CA1 2α-Man^46^). In contrast, epitopes recognized by other MAbs are expressed as discontinuous patchy patterns (e.g. Mab B6.1 3β-Man^35^). It has been shown that the expression pattern of the ligand recognized by MBL is most similar to the expression of epitopes recognized by MAb B6.1^16^.

We cannot currently explain why the B6.1 3β-Man epitope, which drew attention as a protective epitope, has the unique ability to be recognized by MAb EB-CA1 and MBL, molecules known to interact with mannose residues linked in α anomery. However, this dichotomy suggests that such an epitope could be involved in the recognition of yeasts by soluble and membrane receptors, stimulation of the adaptive immune response, and immunomodulation of mucosal and systemic defenses.

Our study suggests that an accumulation of basic and clinical information from the dissection of the anti-mannose immune response is worthwhile for a better understanding of *Candida* pathogenesis and better patient care through the development of vaccines.

## Materials and methods

All methods were carried out in accordance with relevant guidelines and regulations.

### Monoclonal antibodies and antigens

IgM isotype MAbs were selected according to their specificity. MAb EB-CA1, a rat IgM (Bio-Rad Laboratories, France), reacts with an α-(1,2)-mannopentaose as the minimal epitope^36^. Mab 5B2, a rat-mouse hybrid IgM, reacts with β-(1,2)-mannosides with a mannobiose as the minimal epitope^41, 56^. Mab B6.1, a mouse IgM, has been described as specific for a β-(1,2)-mannotriose^42, 57^. Mab EB-A2 (Bio-Rad), reacting with a galactofuranose epitope, was used as the control^49^. Tables 1 and 2 show the MAbs and antigens used in the study.

**Table 1:** Information and references for the antigens used in the study and the technology involved in their analysis.

Antigens/Epitopes	References	Main characteristics	Analytical methods		
Synthetic			Multianalysis profiling	Surface plasmon resonance	Western Blotting/ELISA***
*Biotin Sulfone Tagged Oligomannosides (BSTOs), schematic representation of the epitope*					
Manα-1,2Man			X	X	X
Manα-1,2Manα-1,2Manα-1,2Man			X		
Manβ-1,2Man			X		
Manβ-1,2Manβ-1,2Man	Mat & Meths	Synthesized for the present study	X		
Manβ-1,2Manβ-1,2Manβ-1,2Man			X	X	X
**Native**					
*Phosphopeptidomannan (mannan)* From *C. albicans* VW32 (Serotype A)	^27, 31–33, 55^	Complex cell wall surface polymer. Structure determined by NMR and sequential depolymerization of constitutive oligomannosides used for diagnostic purposes			***
*Phospholipomannan*	^18, 56, 57^	Glycoplipid of the mannoinositolphospho ceramide family Present at cell wall surface Shed in contact with host cells and has immunomodulatory properties			X
From *C. albicans* VW 32 (serotype A)	^19^	Polysaccharide moiety consisting of β-mannosides up to 14 residues			X
From *C. albicans* NIH-B (serotype B)	^20^	Polysaccharide moiety consisting of β-1,2 mannotriose			X
From *C. albicans* BPW17 (serotype A)	^58^	PLM identical to that of VW32. Used for genetic manipulations			X
From *C. albicans* BPW17 depleted in bmt6	^21^	Polysaccharide moiety truncated with a β-mannotriose			X

**Table 2:** Information about antibodies/probes used in the study and technology involved in their analysis.

Probes	Analytical method			References
	Multiple analysis profiling	Surface plasmon resonance	Western blotting	
**Monoclonal antibodies**				
5B2	**X**	**X**	**X**	^34^
EB-CA1	**X**	**X**	**X**	^36^
B6.1	**X**	**X**	**X**	^35^
EB-A2	**X**	**X**	**X**	^59^
**Human polyclonal antibodies**				
Polyclonal antibodies from healthy individuals	**X**			^55, 60, 61^
Polyclonal antibodies from colonized patients	**X**			
Polyclonal antibodies from infected patients	**X**			
**Mannose binding lectin**		**X**		

### Human sera

Human antibodies against BSTOs were characterized in sera taken from three groups of individuals: (1) The first group represents 51 control sera collected from 51 blood donors presenting no disease; (2) The second group consisted of sera collected from 30 ICU patients with systemic *C. albicans* infection, proven by the isolation of *C. albicans* from blood cultures. In this group, sera were taken during the period of serological monitoring from 68 days before to 67 days after *C. albicans* was isolated from the blood; 181 sera were obtained (mean of 6.03 sera per patient); (3) The third group represents 119 sera from 30 patients hospitalized in ICU ward in Besançon University Hospital where colonization by *C. albicans* is followed bi-weekly in order to establish the colonization index (CI). The 30 patients had been hospitalized for > 10 days (mean duration 22 days, maximum 73 days). At admission, all had a corrected colonization index (CCI) of < 0.25. Eight patients were constantly negative for *Candida* colonization, 22 had a CCI ≤ 0.4, and five evolved towards a CCI of > 0.4. A total of 450 mycological samples were examined and the rates of positive isolation of *Candida* were: oropharynx 61%, gastric 30%, tracheal 22%, rectum 17%, and urine 6%. These colonized patients did not have firm clinical evidence of Candida infection.

### Ethics statement

All sera used in this study were obtained from patients monitored at Lille University Hospital. No additional sampling was necessary. As sera were taken from a registered biological collection, patient consent was not required according to French law. Agreement for the establishment of a biological collection of invasive fungal infection samples was obtained from the French Ministry of Education and Research under reference DC2008-642. Institutional review board approval was given by the Comité de Protection des Personnes Nord-Ouest IV, the ethical committee of our institution**.** Sera from colonized patients were collected during a prospective study conducted by one of us which received institutional review approval from the local ethical committee^50^.

### *C. albicans* strains

*C. albicans* serotype A strains consisted of our laboratory reference strain VW32^51^ and BWP17 strain, which was derived from SC5314, the *C. albicans* strain used for sequencing the whole genome^52^. The serotype B strain consisted of NIH B-792^20^. We included a *bmt6Δ* strain, which was constructed from BWP17 by deletion of both alleles for the *BMT6* gene encoding the mannosyltransferase responsible for the addition of the third β-1,2 linked mannose to PLM^21^.

### Western blotting

Strains were grown in YPD medium (1% yeast extract, 2% peptone, 2% dextrose) at 37 °C for 16 h and were extracted using alkaline extraction under reducing conditions^34^. Briefly, cells were incubated on ice in 1.85 M NaOH and 5% β-mercaptoethanol. Proteins and glycoconjugates were then extracted in SDS for 5 min at 100 °C. Extracts were adjusted to the same protein concentration and analyzed by SDS-PAGE on a 5–20% acrylamide gel slab. Membranes were probed with MAbs B6.1 diluted 1:1000, CA1 diluted 1:1000, and 5B2 diluted 1:1000, and then incubated with a 1:1000 dilution of AP-conjugated anti-mouse IgM (B6.1), or anti-rat IgM (CA1 and 5B2).

### Mannan extraction from *C. albicans*

Mannan was prepared from *C. albicans* VW32 grown in bioreactors under standard conditions used for the chemical and immunochemical analysis of this molecule^53^. Quantification of mannan was performed by the sulfuric phenol colorimetric method with a range of standard sucrose solutions. Optical density was measured at 492/620 nm.

### Detection of human antibodies against *C. albicans* mannan

Antibodies to *C. albicans* mannan were detected using the commercially available Platelia *Candida* Ab kit (Bio-Rad, France). The antigen used to coat the ELISA plates is cell wall mannan extracted from *C. albicans* serotype A, strain VW32 (see above), extracted by the method of Kocourek and Ballou^54^. Detection of antibodies was performed according to manufacturer’s instructions.

### Preparation of BSTOs

**Figure 7:**
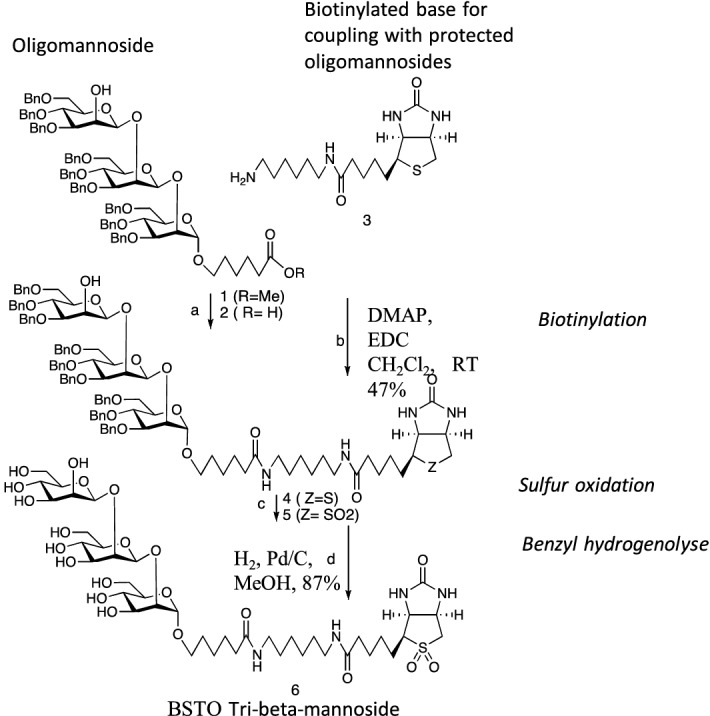
Synthesis of biotinylated β (1 → 2) mannotriose. Compound 6 (tribeta) was prepared in four steps from 1^10^ using coupling and deprotection protocols employed for related di- and tetramannosides^62^. Reagents and conditions: (**a**) NaOH, THF, 60 °C, 98%; (**b**) DMAP, EDC CH_2_Cl_2_, RT 47%; (**c**) meta chloro perbenzoic acid, CH_2_Cl_2_, room temperature, 87%; (**d**) H_2_, Pd/C, MeOH, 87%. In the following Figs. 2, 3, 5, 6 and 7 oligomannosides are designated by acronyms referring to the number of residues and the anomery (i.e. 3β-Man for β-mannotriose) and presented with the color codes already used in studies on oligomannose synthesis (i.e. red for α anomery and green for β anomery^17^), and the main topic of this study, 3β-Man, is represented in darker green.

The synthesis of oligosaccharides and their biotinylation has been described previously^10^, except for mannotriose. The strategy using for the synthesis of biotinylated β (1 → 2) mannotriose is shown in Fig. 7.

### Coupling of BSTOs to fluorescent magnetic beads

Polystyrene fluromagnetic beads were prepared via a four-step procedure as previously described^10^. Briefly, carboxyl functionalized fluromagnetic beads were first activated with N-hydroxysulfosuccinimide and ethylcarbodiimide (Pierce Chemicals Co. Rockford, Ill, USA) to form activated esters. After washing in 50 mM phosphate buffered saline (PBS), pH 7.4, these esters were incubated with avidin (Sigma, Saint Quentin Fallavier, France) for 3 h RT. The beads are then blocked with 50 mM PBS containing 250 mM NH_2_OH for 30 min. After wash in PBS, the beads were coupled with BSTO for 1 h RT and then incubated overnight with 50 mM PBS containing 10% bovine serum albumin. After washing in PBS, the beads were kept at 4° C.

### Quality control of BSTO coupling

For each set of experiments and after coupling BSTOs to magnetic beads, the reactivity of the BSTOs was controlled with a dilution range (1:2000–1:32,000) of biotinylated *Galanthus nivalis* lectin reacting with α-(1,3)-mannose residues or with a panel of polyclonal and monoclonal antibodies followed by detection with streptavidin–phycoerythrin (2 µg/mL). Mixed suspensions of microspheres coated with different BSTOs were incubated with PBS diluted antibodies for 30 min at 37 °C under agitation. After three washes in PBS-1% Tween 20 (PBST) the microspheres were incubated with appropriate anti-immunoglobulins coupled to phycoerythrin (Southern Biotech, USA).

### MAP analysis of BTSO reactivity with sera from patients with invasive candidiasis, ICU colonized patients and controls

Sera were diluted 1:4800 in PBS and incubated for 30 min at 37 °C. After three washes in PBST, the microspheres were incubated with goat anti-human IgG coupled with phycoerythrin (1 µg/mL) (Southern Biotech, USA).

For both procedures, after three washes in PBST the microspheres were re-suspended in sheath fluid in a test tube and the reaction was monitored on a Luminex Lab MAP system 100 (Luminex USA) at 532 nm. The results are expressed as mean fluorescence intensity determined for 100 microspheres of each BSTO identified by its microsphere spectral signature. For human sera, the results were then treated with the "ETALONNAGE" software (University of Lille) and converted into arbitrary units (AU) from calibration curves. Pooled human serum was used as the control standard.

### SPR analysis of BSTO reactivity with anti-carbohydrate MAbs

BIAcore 3000 instrument, BIAevaluation software 3.0, and sensor chip SA (streptavidin) were obtained from BIAcore (GE Healthcare). 5 nM of BSTOs were fixed onto the sensorchip in HBS (HEPES buffered saline) buffer according to the manufacturer’s instructions using NHS/EDC. The immobilization rate was approximately 25–30 response units (RU). 5B2, EB-CA1, B6.1 and EB-A2 MAbs were injected at 250 nM in HBS to 30 μL/min over a 2 min period. The regeneration of sensorship was performed with a 250 mM NaCl/10 mM NaOH buffer. A reference flow cell (i.e. flow cell without BSTOs) was used for each used BSTOs. Quantification of specific binding was obtained from the difference between the ligand and reference response as previously described^10^.

### SPR analysis of BSTO-MBL interactions

With the aim of avoiding non-specific binding induced by non-carbohydrate structures, non-biotinylated oligomannosides were used. rMBL was immobilized onto a CM5 sensorchip (GE Healthcare) in HBS-EP 1X buffer to reach 4000 RU. BSTOs at different concentrations (0, 0.25, 0.5, 1, and 2 mg/mL) were diluted in running buffer composed of HBS-P, CaCl_2_ 5 mM, and surfactant 0.05% and injected in BIAcore 3000 instrument with a flow at 20 µL/min during 4 min. The sensorship was regenerated with 0.25 M EDTA buffer. For each sample, the results are expressed as the difference between the tested flow cell and reference flow cell (flow cell without immobilized rMBL) as previously described^12^. Normalization of results was performed according to the molecular mass of each BSTO.

### Statistical analysis

All statistical analyses were performed using SAS and Med Calc software. Spearman correlation coefficients were calculated to determine the relationship between the reactivity of sera against mannan from *C. albicans* and different BSTOs. The Mann–Whitney U test was used for comparison of antibody levels between different patient groups.

## Supplementary Information

Supplementary Information.
