# Supplementary material for: Dissection of the anti-Candida albicans mannan immune response using synthetic oligomannosides reveals unique properties of β-1,2 mannotriose protective epitopes
Source: Sci Rep. 2021 May 24;11:10825. doi: 10.1038/s41598-021-90402-4 (PMC8144402; doi:10.1038/s41598-021-90402-4)
Supplement: Supplementary file 1 — Supplementary Information. [file 41598_2021_90402_MOESM1_ESM.docx]

**Dissection of the anti-*Candida albicans* mannan immune response using synthetic oligomannosides reveals unique properties of β-1,2 mannotriose protective epitopes**

Boualem Sendid , Karine Lecointe , Mayeul Collot , Pierre-Marie Danzé , Sébastien Damiens , Anne Sophie Drucbert , Chantal Fradin , Jean Pierre Vilcot , Frédéric Grenouillet , Faustine Dubar , Jérôme de Ruyck , Samir Jawhara , Jean Maurice Mallet , Daniel Poulain

**Supplementary information concerning Figure 4.**

The gels were loaded with the same protein concentration of the different extracts of the VW32, NIHB, BWP17 and Δbmt6 strains.  After the transfer, the blots were stained with Ponceau Red before full-length individual strips were cut for incubation in different wells for staining with MAbs B6.1 and EBCA1.  The molecular weight markers (MWM) were carefully reported before analysis.  The limit of the stacking gel is clearly visible on each strip even if only polydispersed glycocongugates are stained.  As well of the alignment of several glycoproteins less glycosylated and thus presenting as more defined lines. This procedure and mode of WB presentation is similar to the previously published papers on PLM in several different journals ^1-11^.  The documents provided in the insert are from reference 21.  Concerning the staining of PLM B with B6.1, a figure published in reference 20 showed a strip stained with MAb B6.1, identical to the one presented in our study (with permission from of Molecular Microbiology, Wiley Author services).

From  Trinel PA, Delplace F, Maes E, Zanetta JP, Mille C, Coddeville B, Jouault T, Strecker G, Poulain D: Candida albicans serotype B strains synthesize a serotype-specific phospholipomannan overexpressing a beta-1,2-linked mannotriose. Mol Microbiol 2005, 58:984-98. With permission of the editor.

**References.**

1 Courjol, F. *et al.* beta-1,2-Mannosyltransferases 1 and 3 Participate in Yeast and Hyphae O- and N-Linked Mannosylation and Alter Candida albicans Fitness During Infection. *Open Forum Infect Dis* **2**, ofv116, doi:10.1093/ofid/ofv116 (2015).

2 Fradin, C. *et al.* Beta-1,2 oligomannose adhesin epitopes are widely distributed over the different families of Candida albicans cell wall mannoproteins and are associated through both N- and O-glycosylation processes. *Infect Immun* **76**, 4509-4517, doi:10.1128/IAI.00368-08 (2008).

3 Gangneux, J. P. *et al.* Clinical Impact of Antifungal Susceptibility, Biofilm Formation and Mannoside Expression of Candida Yeasts on the Outcome of Invasive Candidiasis in ICU: An Ancillary Study on the Prospective AmarCAND2 Cohort. *Front Microbiol* **9**, 2907, doi:10.3389/fmicb.2018.02907 (2018).

4 Mille, C. *et al.* Identification of a new family of genes involved in beta-1,2-mannosylation of glycans in Pichia pastoris and Candida albicans. *J Biol Chem* **283**, 9724-9736, doi:10.1074/jbc.M708825200 (2008).

5 Mille, C. *et al.* Members 5 and 6 of the Candida albicans BMT family encode enzymes acting specifically on beta-mannosylation of the phospholipomannan cell-wall glycosphingolipid. *Glycobiology* **22**, 1332-1342, doi:10.1093/glycob/cws097 (2012).

6 Poulain, D., Slomianny, C., Jouault, T., Gomez, J. M. & Trinel, P. A. Contribution of phospholipomannan to the surface expression of beta-1,2-oligomannosides in Candida albicans and its presence in cell wall extracts. *Infect Immun* **70**, 4323-4328, doi:10.1128/iai.70.8.4323-4328.2002 (2002).

7 Trinel, P. A., Cantelli, C., Bernigaud, A., Jouault, T. & Poulain, D. Evidence for different mannosylation processes involved in the association of beta-1,2-linked oligomannosidic epitopes in Candida albicans mannan and phospholipomannan. *Microbiology (Reading)* **142 ( Pt 8)**, 2263-2270, doi:10.1099/13500872-142-8-2263 (1996).

8 Trinel, P. A. *et al.* Candida albicans serotype B strains synthesize a serotype-specific phospholipomannan overexpressing a beta-1,2-linked mannotriose. *Mol Microbiol* **58**, 984-998, doi:10.1111/j.1365-2958.2005.04890.x (2005).

9 Trinel, P. A., Faille, C., Jacquinot, P. M., Cailliez, J. C. & Poulain, D. Mapping of Candida albicans oligomannosidic epitopes by using monoclonal antibodies. *Infect Immun* **60**, 3845-3851, doi:10.1128/IAI.60.9.3845-3851.1992 (1992).

10 Trinel, P. A., Lepage, G., Jouault, T., Strecker, G. & Poulain, D. Definitive chemical evidence for the constitutive ability of Candida albicans serotype A strains to synthesize beta-1,2 linked oligomannosides containing up to 14 mannose residues. *FEBS Lett* **416**, 203-206, doi:10.1016/s0014-5793(97)01205-2 (1997).

11 Trinel, P. A. *et al.* Candida albicans phospholipomannan, a new member of the fungal mannose inositol phosphoceramide family. *J Biol Chem* **277**, 37260-37271, doi:10.1074/jbc.M202295200 (2002).
